# Implementing a Personalized Integrated Stepped-Care Method (STIP-Method) to Prevent and Treat Neuropsychiatric Symptoms in Persons With Dementia in Nursing Homes: Protocol for a Mixed Methods Study

**DOI:** 10.2196/34550

**Published:** 2022-06-22

**Authors:** Helma M F Verstraeten, Canan Ziylan, Debby L Gerritsen, Robbert Huijsman, Miharu Nakanishi, Martin Smalbrugge, Jenny T van der Steen, Sytse U Zuidema, Wilco P Achterberg, Ton J E M Bakker

**Affiliations:** 1 Department of Public Health and Primary Care Leiden University Medical Center Leiden The Netherlands; 2 Research Centre Innovations in Care Rotterdam University of Applied Sciences Rotterdam The Netherlands; 3 Department of Primary and Community Care Radboud Institute for Health Sciences Radboud university medical center Nijmegen The Netherlands; 4 Erasmus School of Health Policy & Management Erasmus University Rotterdam The Netherlands; 5 Department of Psychiatric Nursing Tohoku University Graduate School of Medicine Sendai-shi Japan; 6 Department of Medicine for Older People Amsterdam Public Health Research Institute Amsterdam University Medical Centre Amsterdam The Netherlands; 7 Department of Primary and Community Care Radboud university medical center Nijmegen The Netherlands; 8 Department of Primary Care and Elderly Care Medicine University Medical Center Groningen University of Groningen Groningen The Netherlands; 9 Stichting Wetenschap Balans Rotterdam The Netherlands

**Keywords:** dementia, neuropsychiatric symptoms, caregiver, implementation, psychosocial intervention, nursing homes

## Abstract

**Background:**

Neuropsychiatric symptoms occur frequently in many nursing home residents with dementia. Despite the availability of multidisciplinary guidelines, neuropsychiatric symptoms are often inadequately managed. Three proven effective methods for managing neuropsychiatric symptoms were integrated into a single intervention method: the STIP-Method, a personalized integrated stepped-care method to prevent and treat neuropsychiatric symptoms. The STIP-Method comprises 5 phases of clinical reasoning to neuropsychiatric symptoms and 4 stepped-care interventions and is supported with a web application.

**Objective:**

This study aims to identify the facilitators and barriers in the implementation of the STIP-Method in nursing homes.

**Methods:**

A mixed methods design within a participatory action research was used to implement the STIP-Method in 4 facilities of 2 Dutch nursing home organizations. In total, we aimed at participation of 160-200 persons with dementia and expected an intervention fidelity of 50% or more, based on earlier studies regarding implementation of effective psychosocial interventions to manage neuropsychiatric symptoms. All involved managers and professionals were trained in the principles of the STIP-Method and in using the web application. An advisory board of professionals, managers, and informal caregivers in each facility supported the implementation during 21 months, including an intermission of 6 months due to the COVID-19 pandemic. In these 6-weekly advisory board meetings, 2 researchers stimulated the members to reflect on progress of the implementation by making use of available data from patient records and the web application. Additionally, the 2 researchers invited the members to suggest how to improve the implementation. Data analysis will involve (1) analysis of facilitators and barriers to the implementation derived from verbatim text reports of advisory board meetings to better understand the implementation process; (2) analysis of patient records in accordance with multidisciplinary guidelines to neuropsychiatric symptoms: personalized, interdisciplinary, and proactive management of neuropsychiatric symptoms; (3) evaluation of the web application in terms of usability scores; (4) pre- and postimplementation analysis of patient records and the web application to evaluate the impact of the STIP-Method, such as changes in neuropsychiatric symptoms and informal caregiver burden.

**Results:**

We enrolled 328 persons with dementia. Data collection started in July 2019 and ended in December 2021. The first version of this manuscript was submitted in October 2021. The first results of data analysis are expected to be published in December 2022 and final results in June 2023.

**Conclusions:**

Our study may increase understanding of facilitators and barriers to the prevention and treatment of neuropsychiatric symptoms in nursing home residents with dementia by implementing the integrated STIP-Method. The need for well-designed implementation studies is of importance to provide nursing homes with optimal tools to prevent and treat neuropsychiatric symptoms.

**International Registered Report Identifier (IRRID):**

DERR1-10.2196/34550

## Introduction

### Background

Neuropsychiatric symptoms such as depression, anxiety, apathy, agitation, and aggressive behavior are highly prevalent in persons with dementia. The prevalence rates of clinically relevant neuropsychiatric symptoms are over 70% [1,2] and the cumulative 2-year prevalence is about 97% [3]. In persons with dementia, neuropsychiatric symptoms are associated with psychological distress, increased mortality, greater functional impairment, lower quality of life, increased emergency department visits, hospitalizations, and long-term care admissions as well as high caregiver burden [4-9].

Although psychotropic medication is often prescribed to manage neuropsychiatric symptoms in persons with dementia, this type of medication is associated with limited effect and considerable side effects [10,11]. National and international guidelines on the management of neuropsychiatric symptoms recommend psychosocial, personalized, and interdisciplinary interventions for first-line treatment to reduce the inappropriate prescription of psychotropic medication [12-15]. Person-centered care, that is, care that fits wishes, needs, and capabilities of persons with dementia and their (informal) caregivers, is the basic principle in the Dutch guideline “Problem behavior in people with dementia” [15]. Several psychosocial multicomponent interventions have been developed to prevent and treat neuropsychiatric symptoms in persons with dementia and are effective according to the Dutch guideline [15]: Integrative reactivation and rehabilitation (IRR) [16]; Grip on Challenging Behavior (Grip) [17]; and the Stepwise, Multidisciplinary Intervention for Pain and Challenging Behavior in Dementia (STA OP!) [18]. As shown in Table 1, these interventions all consist of a continuous loop of detection; analysis; treatment of physical, cognitive, and psychosocial problems; and evaluation. At the same time, these interventions slightly differ from each other. Multimedia Appendix 1 [12, 16,19-33] includes a detailed description of the 3 methods.

Two recent consecutive audits of quality of care in nursing homes carried out by Dutch Health Inspectorate with an interval of 2 years both showed late, inadequate, or incorrect management of neuropsychiatric symptoms despite the existence of national guidelines and advices based on the first audit [34,35]. A broad analysis of neuropsychiatric symptoms is still insufficiently implemented. As a result, possible interventions are poorly implemented [35]. Although IRR, Grip, and STA OP! have been developed and proven to be effective when actually applied as intended, these programs are not broadly implemented within Dutch nursing homes beyond the research setting [35]. Even though the intervention fidelity of IRR, defined as the adherent and competent delivery of an intervention as set forth in the research plan [36], was rather high (90%) within the studied nursing homes, it showed a limited transferability to nursing homes outside the research setting because of a lack in necessary knowledge and skills. Within Grip, all necessary forms of the program were used in only a small percentage of persons with dementia [37]. The intervention STA OP! was performed in only a small proportion (39%) of persons with dementia [18]. An overview of determined facilitators and barriers to implementation is presented in Table 2. The low level of intervention fidelity of these Dutch programs is in line with results of international studies on intervention fidelity of effective psychosocial interventions to manage neuropsychiatric symptoms. For example, the effective DEMBASE program in Japan was fully implemented in 52% of persons with dementia [38]. The overall intervention fidelity of Dementia Care Mapping was poor [39]. Only 13% of nursing homes completed the fully protocol to an acceptable level [39].

**Table 1:** Overview of characteristics of 3 effective methods to manage neuropsychiatric symptoms, as described in Bakker et al [16], Zwijsen et al 2014 [17], and Pieper et al 2018 [18].

Characteristics	IRR^a^	Grip^b^	STA OP!^c^
Proactive method (start when admitted to nursing home)	✓		
Reactive method (start when problems are signaled by nursing staff)		✓	✓
Cyclical process (detection, analysis, treatment, evaluation)	✓	✓	✓
Physical functioning	✓	✓	✓
Assessment and management of pain			✓
Cognitive functioning	✓	✓	✓
Psychosocial functioning	✓	✓	✓
Stepped-care model (stepping up interventions from the least to the most intensive and stepping down, linked to patients’ needs)			✓
Matched-care model (client and therapy are matched, based on intake information about specific problems and patient characteristics)	✓		
Interdisciplinary collaboration	✓	✓	✓
Involvement of informal caregiver	✓	✓	
Treatment of informal caregiver	✓		
Standard involved disciplines	Nurse, elderly care physician, clinical psychologist, social worker	Nurse, psychologist, elderly care physician	Nurse, psychologist, social worker, elderly care physician, occupational therapist, physical therapist
Indicative involved disciplines	For each patient, at least two of the following therapists are involved: music therapist, psychomotor therapist, creative therapist, physical therapist, occupational therapist, speech therapist, dietician	Other disciplines are involved if needed. For example, occupational therapist	N/A^d^

^a^IRR: integrative reactivation and rehabilitation.

^b^Grip: Grip on Challenging Behavior.

^c^STA OP!: Stepwise, Multidisciplinary Intervention for Pain and Challenging Behavior in Dementia

^d^N/A: not applicable.

**Table 2:** Overview of facilitators and barriers of implementation, as described in Zwijsen et al [37], Hakvoort et al [40], and Pieper et al [41]^a^.

	Grip^b^	STA OP!^c,d^
Facilitators for implementation	Support in power, for example, management board of directors Enhanced awareness: positive attitude toward change Group size: 10-15 participants for training sessions	Support in power, for example, presence of persons with a motivational leadership style Enhanced awareness: positive attitude toward change
Barriers for implementation	Staff turnover High workload Involvement in multiple projects or new innovations Canceled meetings Organizational changes Large number of forms to be filled in Lack of digitalized forms Lack of information for informal caregivers	Staff turnover High workload Involvement in multiple projects or new innovations Absence of essential disciplines

^a^Facilitators and barriers were not investigated for integrative reactivation and rehabilitation.

^b^Grip: Grip on Challenging Behavior.

^c^STA OP!: Stepwise, Multidisciplinary Intervention for Pain and Challenging Behavior in Dementia.

^d^Group size was not indicated as a facilitator or a barrier for implementation within STA OP!

Recent studies have shown that interventions should be aimed at both persons with dementia and their informal caregivers. Specific interventions for informal caregivers have long-lasting effects on depression and anxiety symptoms, increase quality of life, and are cost-effective [42]. When implementing a multicomponent care improvement intervention, it is important to understand the implementation process to improve sustainability in clinical practice [12,43,44] to prevent and treat neuropsychiatric symptoms. Although factors for IRR, Grip, and STA OP! have been investigated, nursing homes did not succeed in implementing these methods.

If taken into account the known facilitators and barriers, to what extent will effective methods be implemented? Are there other facilitators and barriers that have not been taken into account? Therefore, the researchers involved in IRR, Grip, and STA OP! (TJEMB, MS, and WPA) and experts on implementation and management of neuropsychiatric symptoms (DLG, JTS, SUZ) collaboratively developed a joint multicomponent intervention, the STIP-Method: a personalized integrated stepped-care method to prevent and treat neuropsychiatric symptoms in persons with dementia in nursing homes, which is compliant with the current Dutch guidelines to manage neuropsychiatric symptoms [15]. In addition to the overarching elements of the existing 3 methods, the stepped-care model was integrated into the STIP-Method. Stepped care can be defined as a staged, evidence-based system comprising hierarchically delivered interventions linked to patients’ needs: from the least to the most intensive, and stepping down or up when needed [45,46]. The integral STIP-Method especially focuses on interdisciplinary collaboration and shared decision making. Shared decision making between professionals and persons with dementia and informal caregivers is of proven importance to achieve real person-centered care [47]. Finally, the STIP-Method is supported by the use of a web application that finds its roots in Sweden: BPSD Care, in which BPSD stands for Behavioural and Psychological Symptoms of Dementia. In Sweden, it forms a nationwide quality registry that is globally acknowledged as an innovation in the psychosocial dementia care program context [48]. Adaptations of the BPSD-registry program have been developed in both Denmark [49] and Japan [50-52]. The use of BPSD Care has been shown to be supportive in significantly reducing neuropsychiatric symptoms, increasing quality of life in persons with dementia [53], and leading to a lower sense of burden among professionals [51].

### Objectives

This study aims to identify the facilitators and barriers in the implementation of the STIP-Method in nursing homes. Based on earlier studies on intervention fidelity of effective psychosocial interventions to prevent and treat neuropsychiatric symptoms [18,37], we hypothesize that the STIP-Method will be delivered according to protocol to 50% or more persons with dementia (intervention fidelity). Additional aims are to evaluate to what extent the STIP-Method is delivered according to protocol (intervention fidelity), to evaluate the impact of the implementation of the STIP-Method on neuropsychiatric symptoms, restraint use, aggression incidents, and use of psychotropic medication. Furthermore, we assess the contribution of the BPSD Care web application to the management of neuropsychiatric symptoms. We expect the study will deliver an in-depth understanding of facilitators and barriers to the management of neuropsychiatric symptoms to positively influence these implementation aspects through (1) emphasis on interdisciplinary collaboration and involvement of informal caregivers, (2) implementation of the web application BPSD Care, and (3) considering the facilitators and barriers regarding the interdisciplinary implementation of Grip and STA OP!

## Methods

### Study Design

We used a mixed methods design within a participatory action research. Principles and processes of participatory action research were used during the implementation of the STIP-Method, and mixed methods were applied through data collection stages (qualitative and quantitative). Participatory action research involves a cyclical process of fact finding, action, and reflection, leading to further inquiry and action for change [54]. This approach has frequently been proven to be effective in involving persons with dementia and informal caregivers and blends research with action [55]. Table 2 shows the enhanced awareness of the method as a facilitating factor in implementing Grip and STA OP! To raise awareness, it is essential that all disciplines experience a sense of urgency and autonomy with regard to management of neuropsychiatric symptoms [37,40,41]. To provide this sense, it is vital to actively involve the target group in the implementation process. Therefore, researchers actively collaborated with professionals, managers, and informal caregivers to support the implementation of the STIP-Method. During implementation, explicit attention was given to the lessons learned from the implementation of IRR, Grip, and STA OP!, so as to understand whether implementation will be improved when these lessons were taken into account. When implementation will not be improved, understanding of the underlying causes is of great importance. Qualitative methods were used to examine facilitators and barriers in the implementation of the STIP-Method. Inductive content analysis was performed using the data directly to define codes and themes, which is further explained in the “Analysis” section [56]. Quantitative methods were used to evaluate the impact of the implementation of the STIP-Method and to assess the contribution of the BPSD Care web application to the management of neuropsychiatric symptoms. Data were collected between July 2019 and December 2021 in the Netherlands with a 6-month intermission due to the COVID-19 pandemic.

### Intervention: The STIP-Method

#### Overview

**Figure 1:**
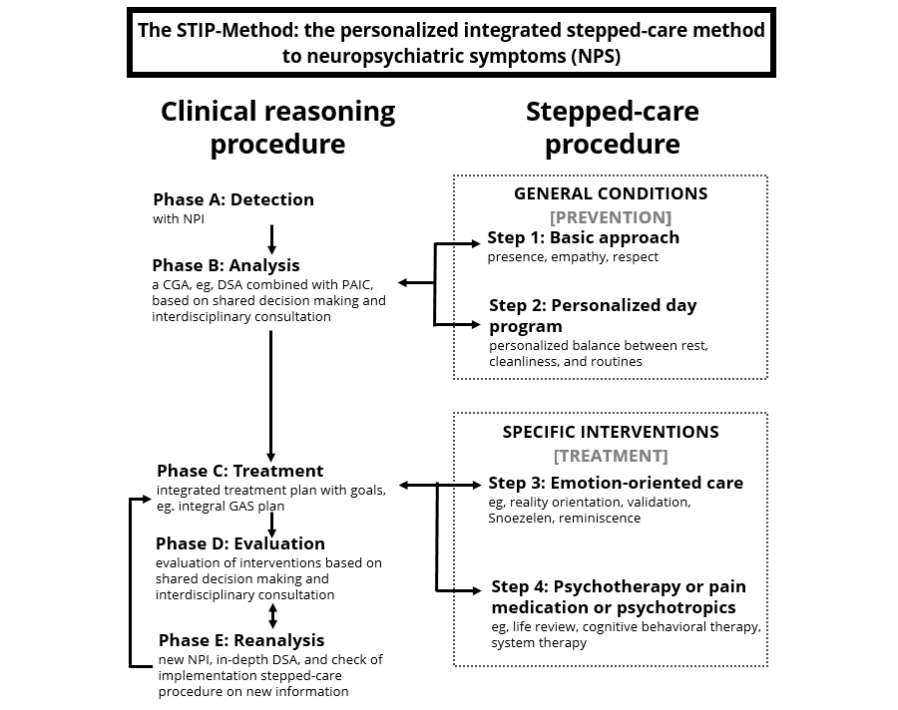
The STIP-Method. CGA: Comprehensive Geriatric Assessment; DSA: dynamic system analysis; GAS: Goal Attainment Scaling; NPI: Neuropsychiatric Inventory; PAIC: Pain Assessment in Impaired Cognition.

The STIP-Method consists of 2 types of procedures: clinical reasoning comprising 5 phases, and a stepped-care procedure comprising 4 interventions (Figure 1). The intervention is supported with the BPSD Care web application to facilitate the clinical reasoning procedure.

#### Clinical Reasoning Procedure

The clinical reasoning procedure is made up of 5 phases (A, B, C, D, and E). Phase A involves identifying and assessing neuropsychiatric symptoms using the Neuropsychiatric Inventory (NPI) [33]. In addition, activities of daily living are assessed via the Barthel Index [57,58], cognitive functioning with the Mini-Mental State Examination (MMSE) [59], and pain perception via the Pain Assessment in Impaired Cognition (PAIC) [60]. In phase B, the analysis phase, factors behind specific behavior are explored based on an extended biography together with the person with dementia or informal caregiver. A broad needs assessment is performed that focuses on basic needs, pain, and physical and psychosocial needs, for example, based on the 7 domains of the Dynamic System Analysis: biology, cognition, personality characteristics, emotional aspects, communication, social context, and life history [61,62]. In phase C, treatment, an integrated treatment plan comprising all involved disciplines is drawn up and carried out. This plan incorporates the relevant themes, goals to be achieved, and interventions to be used per discipline. Subsequently in phase D the integral treatment plan is evaluated in an interdisciplinary manner, with consideration for perceptions of both persons with dementia and their informal caregivers. If the interdisciplinary team concludes that goals in the integral treatment plan are not achieved, an in-depth reanalysis will follow in phase E. For example, when an NPI score does not decrease after the intervention, the patient’s symptoms are assumed to persist. In case of new aspects, the 5 phases of the clinical reasoning process are run through again.

#### Stepped-Care Procedure

Within the stepped-care approach, 4 steps (1-4) of increasing intensity of interventions are distinguished [63,64]. Based on the broad analysis, potentially suitable interventions are examined. Steps 1 and 2 are general conditions for all persons with dementia. Step 1 concerns an appropriate basic approach with presence, empathy, and respect. In step 2 the focus is on a tailor-made daily program that takes into account concrete preferences, hobbies, and activities of persons with dementia based on an in-depth biography. If the effect of these steps is insufficient, specific interventions of steps 3 and 4 can be applied. These steps can also follow immediately after phase B, the analysis phase. In step 3 emotion-oriented care is used to support persons with dementia in coping with the cognitive, emotional, and social consequences by connecting to their individual abilities and subjective experience. Included methods are reality orientation, reminiscence, Powerless in Daily Living (PDL care), and ‘Snoezelen’ (a method to actively stimulate the senses of hearing, touch, vision, and smell in a resident-oriented, nonthreatening environment [65]). PDL care is a type of demand-oriented care that is given multidisciplinary, whereby tools and methods from occupational therapy and physiotherapy are integrated into the care procedures of nurses and therapists [66]. Step 4 refers to a personalized form of (a selection of) 11 available psychotherapeutic interventions from the IRR program focusing on persons with dementia and informal caregivers [16]. Multimedia Appendix 1 includes a detailed description of these psychotherapeutic interventions. It is a common misconception that persons with mild to severe dementia cannot be treated. Of course the cognitive status of a person with dementia plays a role in determining appropriate psychotherapeutic interventions.

#### BPSD Care Web Application

The web-based BPSD registry is translated into Dutch and is launched under the title “BPSD Care web application.” This web application supports the process of clinical reasoning by providing a visualization of longitudinal change in neuropsychiatric symptoms to inform interdisciplinary decision making [38]. Visual feedback to motivate professionals was suggested to be a vital facilitator for implementation in Japan [38]. The registry relies on outlining the frequency, severity, and emotional burden of neuropsychiatric symptoms using the NPI scale (phase A), providing a comprehensive checklist for possible causes of neuropsychiatric symptoms and various scales (MMSE, Barthel Index, and PAIC) to better explore the factors behind specific behavior (phase B). Furthermore, the registry offers evidence-based care plan proposals to reduce neuropsychiatric symptoms and supports monitoring results of the employed interventions in a convenient way. The web application is used adjacent to the regular patient records in nursing home organizations.

### Setting

The STIP-Method was implemented in 4 facilities from 2 Dutch nursing home organizations. Implementation lasted 21 months (from October 1, 2019, to July 1, 2021), including an intermission of 6 months due to the COVID-19 pandemic. In 2017 and 2018, professionals and management employed in these 2 nursing home organizations were trained in step-by-step management of neuropsychiatric symptoms using the stepped-care method. Although the board, management, and professionals of both nursing home organizations indicated that professionals had improved their knowledge about neuropsychiatric symptoms after training, they indicated that they still inadequately involve persons with dementia and informal caregivers in the treatment process. Both organizations recognized the necessity to improve management of neuropsychiatric symptoms and consequently, the STIP-Method was implemented in these 2 organizations.

### Implementation

#### Training

All managers and professionals of the 4 nursing homes were trained in the principles of the STIP-Method. The training course guided (1) neuropsychiatric symptoms and determining underlying factors in persons with dementia; (2) coordination between professionals and collaboration with persons with dementia and informal caregivers; (3) the use of web application BPSD Care; and (4) implementation of agreed interventions with an emphasis on stepped-care procedure steps 1 and 2. Interventions from steps 3 and 4 were discussed briefly. The training was provided by involved researchers during 4 sessions of 3 hours and was organized at their own facility. A group size between 10 and 15 participants for training sessions was maintained (facilitating factor of the Grip study) [40]. In-depth training is necessary to master stepped-care procedure steps 3 and 4. Participants were encouraged by the researchers to attend an available elaborate training course for steps 3 and 4. This course was provided beyond the scope of this study.

#### Advisory Boards

The implementation of the STIP-Method was supported by means of (1) advisory boards, (2) collective advisory board meetings, and (3) a project group. Based on the identified facilitators in the Grip and STA OP! studies [40,41], the advisory boards consisted of, on average, 8 members: a psychologist, an elderly care physician, a manager, 2 informal caregivers, and 2 or 3 members of the nursing staff. The advisory board meetings were held in each facility once every 6 weeks and lasted 90 minutes. Progress of implementation was discussed based on data from patient records and the BPSD Care web application. The meetings were moderated by 2 researchers (TJEMB and a researcher outside the study group) to facilitate the process of identifying facilitators and barriers. The agenda for the meetings was determined by the members of the advisory boards, enabling them to discuss what was important to them to further improve the implementation. In addition, a collective advisory board meeting was planned every 24 weeks for the 4 advisory boards to learn from each other’s experiences. Furthermore, a project group of professionals, managers, and board members of each organization met every 12 weeks to discuss progress and to make adjustments at organizational level where necessary (facilitator of the Grip Study [40]).

#### Support by Two Researchers

During the whole intervention period, 2 researchers (1 implementation specialist and 1 STIP-Method content specialist [TJEMB]) supported and stimulated participants of the advisory boards to reflect on available data from patient records and the web application BPSD Care. Additionally, researchers encouraged participants of advisory boards to seek for ways to increase implementation.

### Study Population

To identify differences in implementation aspects in and between nursing home organizations, both organizations have chosen 2 participating facilities. On average, a nursing home facility houses 40-50 persons with dementia. Taking into account the number of beds and turnover of nursing home residents, 160-200 persons with dementia were expected to be included within the 4 facilities.

### Recruitment

Within each nursing home facility, managers were asked to send an invitation letter, a participant information letter, and a consent form to the legal representatives of persons with dementia. The representatives were asked to allow researchers to access the patient records. Representatives could give permission by signing and returning the written informed consent form to the nursing home in a prestamped envelope. After 4 weeks, a reminder was sent to the managers to motivate their team members to contact representatives about the informed consent forms. Participants had the right to withdraw from participation at any time. No financial incentive to participate was provided. Besides recruitment of persons with dementia, each participating nursing home facility had to form an advisory board. Managers had the freedom to decide whom to invite to the advisory board meetings. Informal caregivers received financial compensation because of the effort required to attend the meetings.

### Inclusion and Exclusion Criteria

Persons with dementia were included if they met the following criteria: (1) dementia diagnosis; and (2) residing on psychogeriatric wards of nursing homes. Informal caregivers had to be able to understand and communicate in Dutch.

### Data Collection

#### Methods to Identify Facilitators and Barriers in the Implementation of the STIP-Method

During the advisory board meetings, participants were invited to share suggestions for facilitators and barriers at organizational and facility levels. Additionally, board members and managers were interviewed after implementation in order to challenge them to reflect on their role during the implementation process and to identify their perceived facilitators and barriers. Finally, after the implementation period, we collected experiences from participants of advisory board groups by conducting an online survey to assess the contribution of advisory board groups toward the implementation of the STIP-Method. Topics were frequency, composition of the advisory board meetings, and usefulness of the meetings.

#### Instruments to Assess Intervention Fidelity

To evaluate if the STIP-Method was implemented in a personalized, interdisciplinary, and proactive manner, we assessed all patient records and registries in the BPSD Care web application at the start and the end of the implementation. Consideration was given to the availability and date of completion of 5 phases of clinical reasoning and 4 stepped-care interventions. Whether the care was personalized was evaluated by checking if an appropriate basic approach with presence, empathy, and respect (step 1) was used and if a tailor-made daily program was drawn up (step 2). With regard to interdisciplinary collaboration, we assessed whether and to what extent an integral treatment plan was applied. During a consensus meeting of the involved researchers of IRR, Grip, and STA OP!, it was decided to define proactive implementation as 2 weeks after admission to the nursing home, based on earlier research and previous experiences [16]. Furthermore, a qualitative analysis was carried out on 50 patient records at baseline and 50 after implementation. We used a specific quality standard based on the Dutch multidisciplinary guideline on problem behavior in dementia [15]. With regard to the qualitative analysis, the 9 elements of the STIP-Method were graded using a 4-point scale (missing, insufficient, sufficient, and good). The definitions of these grades are outlined in Table 3. All observations were independently scored by 2 researchers (HMFV and a researcher outside the study group) to monitor interrater reliability.

**Table 3:** Qualitative analysis of patient records.

Definition			Good (=standard)	Sufficient	Insufficient		
Clinical reasoning phases							
	A: Detection		Neuropsychiatric inventory is fully completed Results are discussed in an interdisciplinary manner	Does not fully meet the standard	Does not meet the standard at all		
	B: Analysis		Biography consists of concrete information on physical, psychological, and social domains Biography is up to date Broad analysis includes at a minimum a physical examination, neuropsychological factors, biography, information about personality and contextual factors	Does not fully meet the standard	Does not meet the standard at all		
	C: Treatment		Integral treatment plan (with informal caregiver, psychologist, professionals, and elderly care physician) involves at least physical, psychological, and social domain Attention for informal caregiver aspects within the social domain Focus on factors extracted from broad analysis Measurable treatment goals and interventions	Does not fully meet the standard	Does not meet the standard at all		
	D: Interdisciplinary evaluation: behavior visits^a^, multidisciplinary consultations, and care plan reviews^b^		Evaluation of goals and degree of implementation of actions Information about progress and satisfaction of persons with dementia and informal caregiver is available Appointment for next evaluation is available	Does not fully meet the standard	Does not meet the standard at all		
	E: Reanalysis		Not further defined: reference to phases A and B				
Stepped-care interventions							
		1: Basic approach	Results from broad analysis Describes how real contact, with presence, empathy, and respect, can be made with persons with dementia Is based on the needs of the person with dementia and informal caregiver	Does not fully meet the standard	Does not meet the standard at all		
		2: Personalized day program	Results from broad analysis Fits well with the needs of the person with dementia Concrete preferences, hobbies, and activities are taken into account Consists of concrete actions and activities Easy to find in patient record	Does not fully meet the standard	Does not meet the standard at all		
		3: Emotion-oriented care	Results from broad analysis Responds to underlying needs and causes Easy to find in patient record Drawn up on an interdisciplinary manner	Does not fully meet the standard	Does not meet the standard at all		
		4: Psychotherapeutic interventions	Interventions to target the diagnosed physical function problems Focus on emotional experience, personality, traumatic life experiences, social functioning (including informal caregiver burden)	Does not fully meet the standard	Does not meet the standard at all		

^a^Visits related to neuropsychiatric symptoms and with the presence of at least a psychologist, an elderly care physician, and a registered or practice licensed nurse.

^b^Reviews of the care plan with the presence of an elderly care physician and a registered or practice licensed nurse.

#### Instruments to Assess the Impact of the Implementation of the STIP-Method

During the implementation period, the progress of the NPI scores among persons with dementia was used to assess the impact of the implementation on the level of frequency, severity, and informal caregiver burden of neuropsychiatric symptoms. In addition, the number of freedom-restricting measures, aggression incidents, and the prescription of psychotropic medication were used.

#### Instruments to Assess to What Extent the BPSD Care Web Application Does Contribute to the Facilitation of Clinical Reasoning and the Management of Neuropsychiatric Symptoms

**Figure 2:**
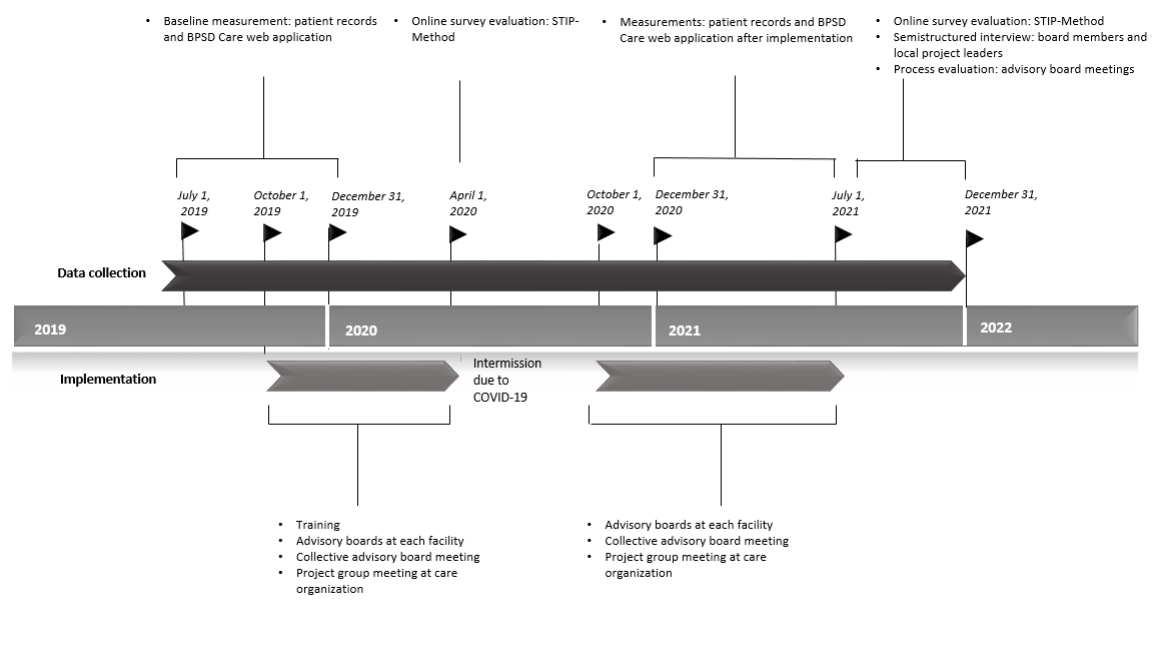
Timeline for implementation and data collection. BPSD: Behavioural and Psychological Symptoms of Dementia.

At baseline and after implementation, all involved professionals were requested to fill in a digital, self-constructed questionnaire to evaluate ongoing implementation of the STIP-Method, including the BPSD Care web application. Participants rated the usability of the web application on items of the System Usability Scale, a validated 10-item questionnaire with a 5-point response scale ranging from strongly disagree (1) to strongly agree (5) that lead to a score between 1 and 100 [67]. Job satisfaction was measured by 7 questions derived from the Dutch employee satisfaction survey from ActiZ (Dutch Association for Health Care Providers in Elderly Care) [68]. The Employee Net Promotor Score was used to summarize caregiver satisfaction and is based on a single question: “How likely is that you would recommend our organization to a friend or colleague?” Participants gave an answer ranging from 0 (not all likely) to 10 (extremely likely). The score is calculated as the percentage of “promotors” (individuals scoring a 9 or 10) minus the percentage of “detractors” (individuals answering 0-6) [69]. To assess the general usability of the STIP-Method, questions regarding 5 phases of clinical reasoning and 4 stepped-care interventions were added with a 5-point response scale ranging from not useful (1) to very useful (5). Furthermore, a multiple-choice question was asked to assess the purpose of using the web application. In addition, verbatim text reports of advisory board meetings were used to assess the contribution of the web application to the management of neuropsychiatric symptoms. An extended overview of the used assessment instruments is shown in Table 4. Figure 2 depicts a timeline for implementation and data collection.

**Table 4:** Overview of concepts, measures, and measurements to assess the implementation of the STIP-Method, a personalized integrated stepped-care method.

Source and assessment			Measurement instrument		Time of measurement		
				Start implementation		End implementation	
**Advisory board meetings**							
	Facilitators and barriers	Advisory boards at each facility^a^					
		Collective advisory board meeting (all 4 facilities)^b^					
		Project group^c^					
**Patient records**							
	Availability and date of completion of 5 phases of clinical reasoning + 4 stepped-care interventions	Quality standard: STIP-Method		✓		✓	
	Quality check patient records with a 4-point scale (good, sufficient, insufficient, and missing)	Quality standard: STIP-Method		✓		✓	
**BPSD^d^ Care**							
	Neuropsychiatric symptoms	Neuropsychiatric Inventory		✓		✓	
	Broad needs assessment	Inventory of causes based on Dynamic System Analysis		✓		✓	
	Cognitive functioning	Mini-Mental State Examination		✓		✓	
	Activities of daily living	Barthel Index		✓		✓	
	Pain	Pain Assessment in Impaired Cognition		✓		✓	
**Pharmacists’ electronic patient records**							
	Medication use	Use of the ATC^e^ classification system on psychotropic medication: antipsychotics (N05A), anxiolytics (N05B), hypnotics (N05C), antidepressants (N06A), anti-dementia medication (N06D), and anti-epileptic medication (N03)		✓		✓	
**Patient records**							
	Demographics	Organization and facility		✓		✓	
		Type of dementia		✓		✓	
		Date of admission to nursing home		✓		✓	
		Demographics: sex, date of birth		✓		✓	
		Restraint use		✓		✓	
		Reported aggression incidents		✓		✓	
**Online survey**							
	Evaluation of the STIP-Method	Short evaluation of ongoing implementation of the STIP-Method, including the BPSD web application. To assess feasibility, satisfaction, job satisfaction		✓		✓	
	Process evaluation advisory board meetings	Evaluation of using advisory board groups. Focusing on frequency, composition, utility, and effects				✓	
**Semistructured interview**							
	Process evaluation of the STIP-Method	Board members and local project leaders				✓	

^a^Every 6 weeks (12 in total).

^b^Every 6 months (4 in total).

^c^Every 3 months (8 in total).

^d^BPSD: Behavioural and Psychological Symptoms of Dementia

^e^ATC: Anatomical Therapeutic Chemical.

### Analysis

Data extracted from patient records were coded with unique identification numbers to guarantee privacy. All analyses will be undertaken at the level of nursing home and persons with dementia. All advisory board meetings were audio-recorded. Verbatim transcription will be done by a researcher (HMFV) with the support of research assistants. The transcripts will be coded using content analysis, facilitated by the qualitative analysis software ATLAS.Ti.9. Two researchers (HMFV and CZ) will code in vivo the first 3 advisory board meetings together to reach clarity about how to code the board meetings consistently. Hereafter, the researchers will discuss any conflicting codes and ambiguous statements to come to an agreement about the final coding scheme. Then, the identified facilitators and barriers will be categorized into main themes.

### Ethics Approval

The study started after being reviewed by the Medical Ethics Committee of the Erasmus University Rotterdam, under file number MEC-2019-0343.

### Dissemination Plan

At the completion of the study, we will present the findings at national and international scientific conferences; professional events for stakeholder group; and at our professional website. Findings will be presented in a summarized form with no identifying information. We will also publish the results in peer-reviewed journals, open access publications, and lay magazines.

## Results

We enrolled 328 persons with dementia. Data collection started in July 2019 and ended in December 2021. The first version of this manuscript was submitted in October 2021. The first results of data analysis are expected to be published in December 2022 and final results in June 2023.

## Discussion

### Relevance

This protocol describes a mixed methods study within a participatory action research to implement the STIP-Method to manage neuropsychiatric symptoms in persons with dementia in nursing homes. The STIP-Method is developed from 3 already proven effective methods to manage neuropsychiatric symptoms. Previous research on the implementation of these 3 methods showed a low level of intervention fidelity. A better understanding of the implementation process is necessary to improve sustainability in clinical practice to improve the management of neuropsychiatric symptoms [12,43,44]. We expect the study will deliver an in-depth understanding of facilitators and barriers to the management of neuropsychiatric symptoms to positively influence these factors. Although not an effect study, it aims to measure impact in real-life care settings related to the intervention fidelity.

### Strengths and Limitations

A strength of this study is that the STIP-Method is more comprehensive, as it uses the key elements from the underlying effective interventions. Furthermore, we aimed to gain insight into the best manner of implementation by using an advisory board of, among others, informal caregivers. A possible limitation is a potential tension in nursing homes between urgency as recognized in the participating organizations and the increased difficulty to implement an intervention with more elements than the already known stepped-care interventions. In addition, BPSD Care was used as an application adjacent to patient records. Within the Grip study, managers prior to implementation indicated a tool adjacent to patient records as a barrier [40]. However, within the Grip study, using 2 systems did not cause any problems. The lack of a connection between systems may be a barrier in our study.

### Conclusions

We anticipate that our results can be used to improve the intervention fidelity of multicomponent interventions to prevent and treat neuropsychiatric symptoms in persons with dementia. These improvements may enhance quality of life for persons with dementia and their informal caregivers and may improve job satisfaction and the attractiveness of their profession.

## Appendix

Multimedia Appendix 1 Description of existing effective methods: 1. Integrative reactivation and rehabilitation (IRR), 2. Grip on challenging behavior (Grip) and 3. Stepwise, Multidisciplinary Intervention for Pain and Challenging Behavior in Dementia (STA OP!); similarities and differences.

## Abbreviations

BPSD: Behavioural and Psychological Symptoms of Dementia
Grip: Grip on Challenging Behavior
IRR: integrative reactivation and rehabilitation
MMSE: Mini-Mental State Examination
NPI: Neuropsychiatric Inventory
PAIC: Pain Assessment in Impaired Cognition
STA OP!: Stepwise, Multidisciplinary Intervention for Pain and Challenging Behavior in Dementia

## Data Availability

The data sets generated and analyzed during this study will be available from the corresponding author on reasonable request. We will use the DataverseNL, a publicly accessible data repository platform.
