# Supplementary material for: Implementing a Personalized Integrated Stepped-Care Method (STIP-Method) to Prevent and Treat Neuropsychiatric Symptoms in Persons With Dementia in Nursing Homes: Protocol for a Mixed Methods Study
Source: JMIR Res Protoc. 2022 Jun 22;11(6):e34550. doi: 10.2196/34550 (PMC9260522; doi:10.2196/34550)
Supplement: Multimedia Appendix 1 [file resprot_v11i6e34550_app1.docx]

**Multimedia Appendix 1.** Description of existing effective methods: 1. Integrative reactivation and rehabilitation (IRR), 2. Grip on challenging behavior (Grip) and 3. Stepwise, Multidisciplinary Intervention for Pain and Challenging Behavior in Dementia (STA OP!); similarities and differences.

1. Integrative reactivation and rehabilitation (IRR)

IRR is a clinical program that applies a proactive and personalized, integrative, and psychiatric or psychotherapeutic perspective to both persons with dementia and informal caregiver, and is executed by a multidisciplinary team (see Figure S1). The IRR program has a duration of 13 weeks. In addition to usual multidisciplinary nursing home care, including psychotropic medication treatment, IRR consists of personalised, integrative psychotherapeutic interventions to treat multiple psychiatric symptoms of persons with dementia, and family therapy for the informal caregiver. Neuropsychiatric symptoms are measured using the Neuropsychiatric Inventory (NPI)[33]. Furthermore, cognitive and somatic functioning are optimized. In the first phase of the method possible function disorders of person with dementia and informal caregiver will be identified for each person with dementia. These function disorders are systematically ordered according to six dimensions: (1) emotion; (2) personality; (3) life events; (4) psychosocial functioning; (5) cognitive functioning; (6) somatic function disorders. A personal package of interventions is composed and recorded in an interdisciplinary goal attainment plan for each person with dementia and informal caregiver based on these six dimensions [19, 20]. The following psychotherapeutic interventions are available and written down in specific guidelines for each discipline: (1) diagnostic assessment; (2) counselling; (3) life review; (4) interpersonal therapy; (5) cognitive behavior therapy; (6) behavior therapy; (7) support in accepting behavior and minimizing negative effects; (8) regression approach, temporarily accepting regression behavior; (9) rehabilitation;(10) support from social worker on discharge; (11) psychoeducation; and (12) system therapy [19]. The multidisciplinary IRR team consists of, among others, a nursing team, elderly care physician, clinical psychologist, social worker, music therapist, psychomotor therapist, creative therapist, physiotherapist, speech therapist, and a dietician[19]. Interventions are mainly provided in a group of persons with dementia, but individually when necessary. Progress of the person with dementia and informal caregiver is monitored weekly, guided by the method of standardized goal attainment scaling (GAS) [16].


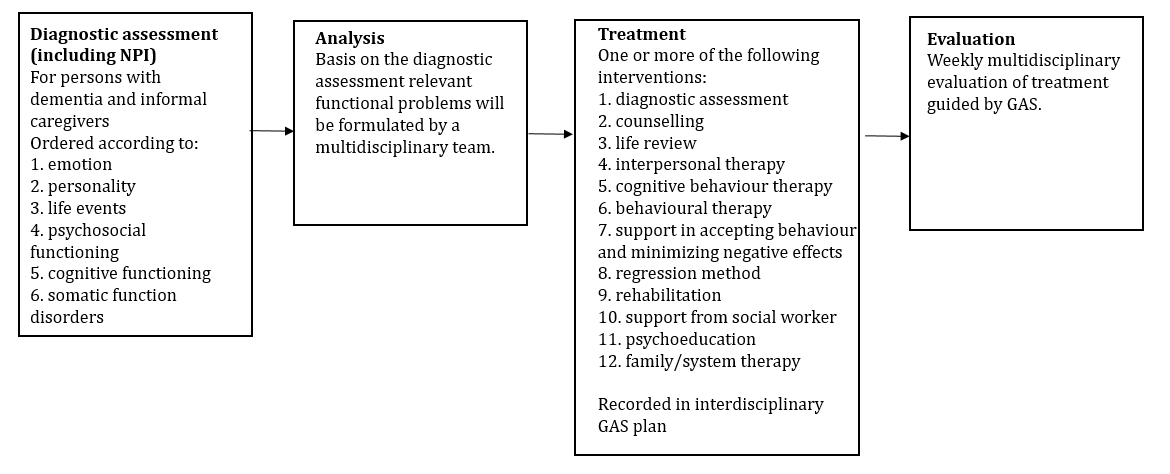


Figure S1. Intervention Integrative reactivation and rehabilitation (IRR)

1. Grip on Challenging behavior

Grip on Challenging behavior is a care program that consists of 4 steps: detection, analysis, treatment and evaluation (see Figure S2) [21]. The goal of the first step, detection, is to detect signs of neuropsychiatric symptoms at an early stage. The nursing staff (i.e., registered nurses, certified nursing assistants, and nurse aides) initiate this step, and other disciplines can support it by emphasizing the importance of early detection. The tool used is the NPI-Q, a shortened version of the Neuropsychiatric Inventory [22]. The second step, analysis of neuropsychiatric symptoms, starts when neuropsychiatric symptoms are identified. The goal of this step is to get a clearer picture of the behavior and its possible causes. Analysis is started by the nursing staff and followed up by physician, psychologist or both disciplines. The third step, treatment, concerns making a treatment plan that includes a clear treatment goal. A meeting is arranged between the involved disciplines to discuss the results of the analysis. The psychologist or elderly care physician is responsible for this step. The choice of interventions is based on hypotheses of what causes the behavior, individual preferences of persons with dementia, and availability of treatment options in the nursing home. Step four refers to structural evaluation of results of the intervention with the involved disciplines. The psychologist or elderly care physician is responsible for this step. A decision can be made about which steps should be taken next: stop or continue treatment (if the treatment goal was achieved after intervening), change treatment, or revise the analysis (if the treatment goal was not achieved after all planned actions were undertaken) [21].


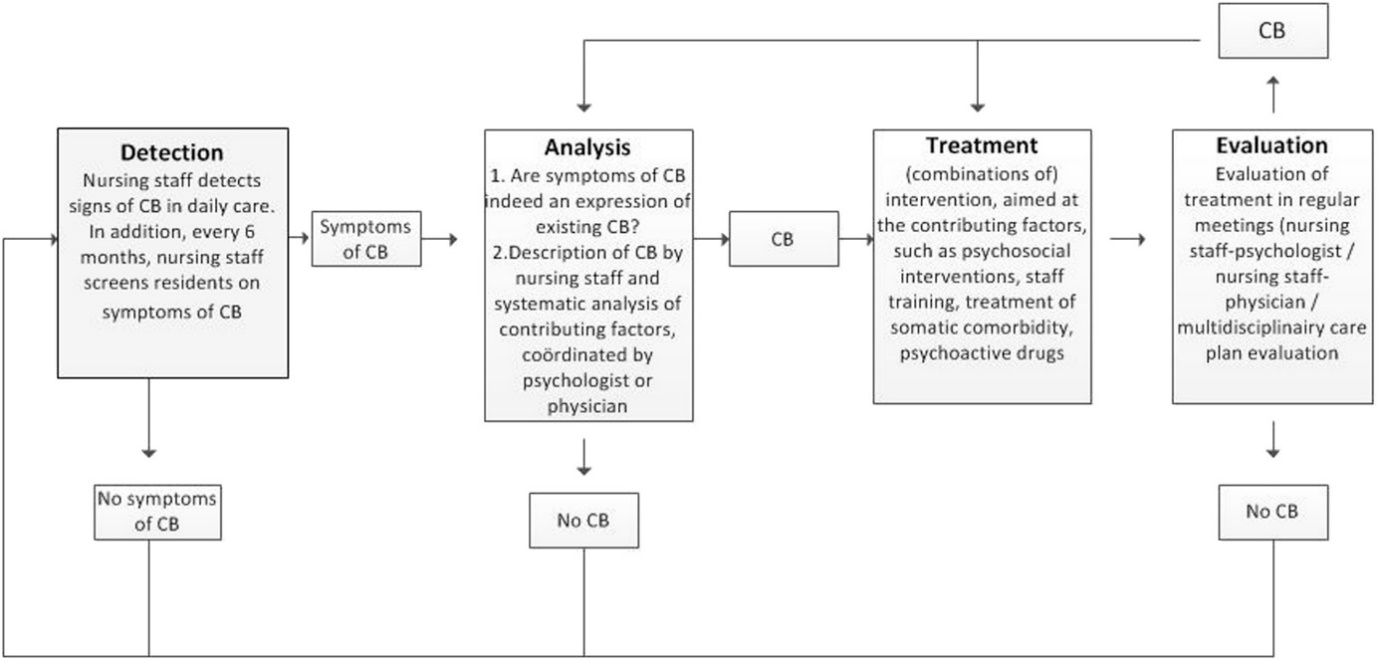

Figure S2. Components of the care programme Grip on challenging behaviour (CB)

Note: This Figure was originally published in *BMC Health Services Research.* 2011; 11(41). Zwijsen SA, Smalbrugge M, Zuidema SU, Koopmans RTCM, Bosman JE, van Tulder MW, et al. Grip on challenging behavior: a multidisciplinary care programme for managing behavioral problems in nursing home residents with dementia. Study protocol.

1. STA OP!

STA OP! is the Dutch version of the serial trial intervention (STI) developed in the United States to address the problems in the assessment and management of pain and challenging behavior in persons with dementia [23-26]. It is based on the theoretical framework of ‘unmet needs’, in which behavior can be seen as a way for cognitively impaired persons to express their physical and affective needs [12, 27]. The intervention uses a stepped-care protocol, i.e., if the assessment is negative or if targeted interventions fail to reduce neuropsychiatric symptoms in one step, one moves to the next step. Neuropsychiatric symptoms were measured with the Dutch version of the Neuropsychiatric Inventory-Nursing Home Version (NPI-NH) [28, 29] and with the Dutch version of the Cohen-Mansfield Agitation Inventory (CMAI) [30]. Symptoms of pain were measured with the Pain Assessment Checklist for Seniors (PAC-SLAC-D) [31] and with the pain scale of the Dutch version of the Minimum Data Set of the Resident Assessment Instrument (MDS-RAI) [32]. In case of neuropsychiatric symptoms, the nursing team and therapists together formulate a clear description of the targeted behavior. They define the target behavior, its expression and in what situation the behavior is challenging. Then the nurse moves to the next step (0). In this step, the nurse assesses whether basic physical care needs are fulfilled, for instance hunger, thirst, a need for glasses or toileting. If the assessment is negative or if treatment fails to decrease symptoms, the nurse moves to the next step (step 1). The first step is to perform a physical needs assessment that focuses on probable causes of neuropsychiatric symptoms related to pain and discomfort. The second step implies performing a needs assessment that focuses on affective needs of persons with dementia with guidance from the psychologist. The third step concerns treatment consisting of non-pharmacological comfort interventions i.e. soothing, supportive verbal communication and sensory stimulation. The fourth step includes a trial of analgesics. Step five refers to consultation of other healthcare professionals or practitioners, or a trial of psychotropic medication (see Figure S3).


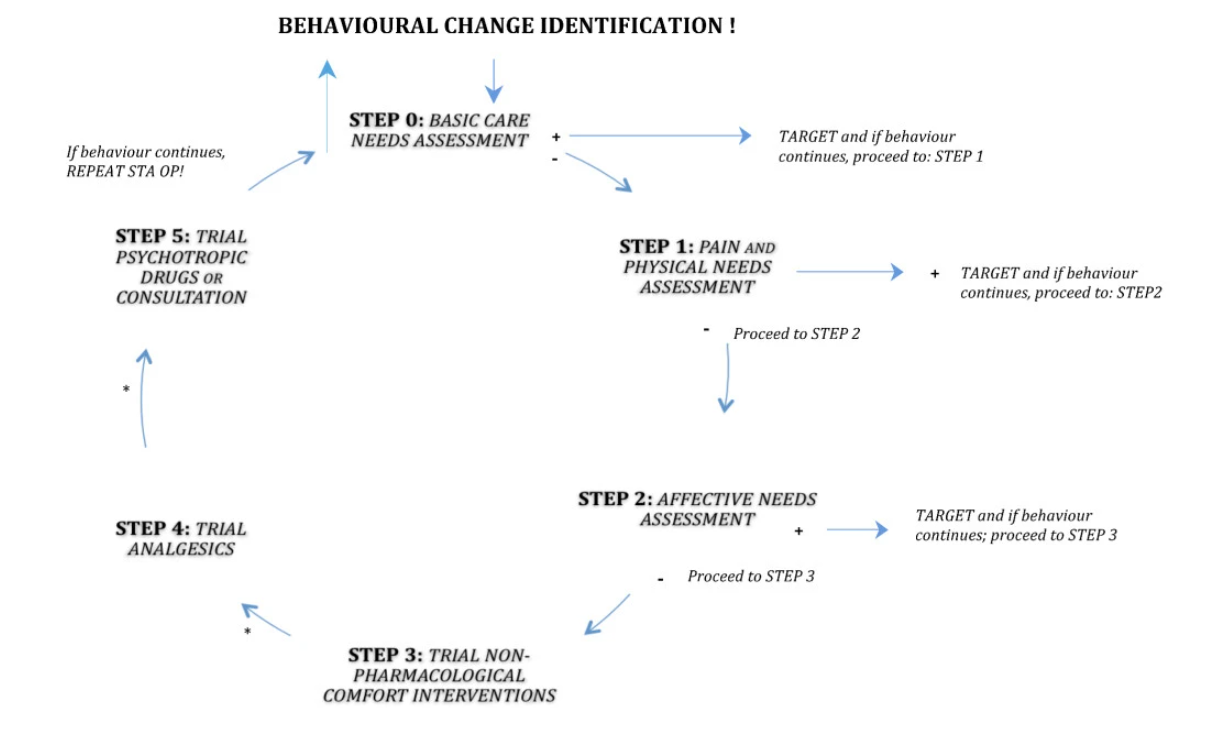


Figure S3. The STI and STA OP! intervention

Note: This Figure was originally published in *BMC Geriatrics.* 2011; 11(12). Pieper MJC, Achterberg WP, Francke AL, van der Steen JT, Scherder EJA, Kovach CR. The implementation of the serial trial intervention for pain and challenging behavior in advanced dementia patients (STA OP!): a clustered randomized controlled trial.
